# Improvement in Memory and Brain Long-term Potentiation Deficits Due to Permanent Hypoperfusion/Ischemia by Grape Seed Extract in Rats

**Published:** 2013-09

**Authors:** Alireza Sarkaki, Maryam Rafieirad, Seyed Ebrahim Hossini, Yaghoub Farbood, Fereshteh Motamedi, Seyed Mohammad Taghi Mansouri, Bahareh Naghizadeh

**Affiliations:** 1 Physiology Research Center and Medicinal Plants Research Center, Ahvaz Jundishapur University of Medical Sciences, Ahvaz, Iran; 2 Department of Biology, Sciences Faculty, Islamic Azad University, Izeh Branch, Izeh, Iran; 3 Department of Biology, Sciences & Researches Branch, Islamic Azad University, Fars, Iran; 4 Department of Physiology and Physiology Research Center, Ahvaz Jundishapur University of Medical Sciences, Ahvaz, Iran; 5 Iranian Neurosciences Research Network and Neurosciences Research Center, Shahid Beheshti University of Medical Sciences, Tehran, Iran; 6 Department of Pharmacology, Physiology Research Center, Ahvaz Jundishapur University of Medical Sciences, Ahvaz, Iran

**Keywords:** Grape seed extract, Hypoperfusion, Ischemia, LTP, Memory, Rat

## Abstract

***Objective(s):*** Cerebral hypoperfusion/ischemia (CHI) is a neurological disease where impaired hippocampus electrical activity and cognition caused by a serial pathophysiological events. This study aimed to evaluate the effects of chronic oral administration of grape seed extract (GSE) on passive avoidance memory and long-term potentiation (LTP) after permanent bilateral common carotid arteries occlusion (2CCAO) in male adult rats.

***Materials and Methods:*** Thirty-two adult male Wistar rats were randomly divided into: 1) Sham+Veh, 2) Isch+Veh, 3) Sham+GSE, 4) Isch+GSE. In order to make 2CCAO as an animal model of CHI, carotid arteries were ligatured and then cut bilaterally. To evaluation of passive avoidance memory, step-down latency (STL) was measured and LTP was recorded from hippocampal dentate gyrus (DG) after high frequency stimulation (HFS) in all rats.

***Results:*** We found that memory was significantly impaired in rats after CHI (*P*<0.001) concomitant with hippocampal LTP inhibition (*P*<0.05, *P*<0.01 for LTP_1 _and LTP_48 _respectively). GSE treatment significantly improved memory impairment and increased hippocampal LTP in rats with 2CCAO.

***Conclusion:*** Our results in present study suggest that GSE exhibits therapeutic potential for short-and long-term memories as well as LTP in DG, which is most likely related at least in part to its antioxidative and free radical scavenging actions.

## Introduction

It has been widely accepted that chronic cerebral hypoperfusion induces memory deficits and oxidative stress damage in neuronal tissues and cells, partially due to the generation of reactive oxygen species (ROS) and reactive nitrogen species (RNS) (1-2). Global cerebral ischemia in rodents is an established model in experimental research on cerebral ischemia, morphologically characterized as a selective neuronal damage in the hippocampus, striatum and cortex. Based upon these results, it has been analyzed whether substances which interact with the pathophysiological processes reduce the ischemic neuronal damage (3). Cerebral ischemia resulted from low oxygen and glucose supply, evidently decreases the formation of ATP (4-5). Damage to brain tissue resulting from cerebral ischemia is a major cause of adult disability that can lead to cognition problems, seizures and death (6-7). 

Although ischemia disrupts cerebral blood flow (CBF), it can lead to brain injury from influx of neutrophils and increase ROS, cerebral edema, and hemorrhage (8). Permanent bilateral common carotid arteries occlusion (2CCAO) in rats has been used as an animal model of chronic cerebral hypoperfusion ischemia (CHI) (9-10). More recently, it has been reported that pathological events such as ischemia and energy deprivation may also induce long-term changes in excitatory synaptic transmission in hippocampal pyramidal neurons (11).

GSE is a rich source of polyphenolic compounds, such as catechin, epicatechin, and dimeric and tetramer Proanthocyanidin (12). The beneficial effects of grape seed polyphenols are due to their free radicals scavenging capability. It has been shown that the antioxidant activity of grape seed and grape juice polyphenols is superior to other well-known antioxidants, such as vitamin C, vitamin E and beta-carotene (13-16). Antioxidants are potential candidates for prevention or treatment of disorders involving oxidative stress (11). Antioxidant activities might be a major contributory factor to the role of grape seed polyphenols in ischemic injuries and brain inflammation (17-18). Some reports have shown that active constituents in grape seed extract such as Proantocyanidin inhibits glutamate-induced cell death through inhibition of calcium signals and nitric oxide formation in cultured rat hippocampal neurons (44). In addition, Charradi *et al* demonstrated that GSE could alleviate high fat diet-induced inhibition of glutamine synthetase activity, acting as a glutamine-glutamate GABA cycle modulator in the brain (45).

The current study aimes to investigate the effects of chronic oral administration of GSE on passive avoidance memory deficit and hippocampus dentate gyrus (DG) long-term potentiation (LTP) inhibition induced by permanent 2CCAO as an animal model of CHI.

## Materials and Methods

***Animal groups***

Thirty-two adult male Wistar rats (220±30 g) were individually housed in standard cages under controlled room temperature (20±2°C), humidity (50-55%) and 12:12 hr light/dark cycle. Access to food and water was *ad libitum*. All experiments were under control of the Local Ethics Committee for the Purpose of Control and Supervision of Experiments on Laboratory Animals. Ten days after remaining in the laboratory, rats were randomly divided into four groups, 8 in each: group 1: Sham+Veh (operation was carried out but the common carotid arteries remained intact and received the same volume of GSE vehicle, 28 days, P.O.); group 2: Sham+GSE (operation was done but the common carotid arteries remained intact and received GSE 100 mg/kg, 28 days, P.O.) (19-20); group 3: Isch+Veh (the common carotid arteries were cut bilaterally and received the same volume of GSE vehicle, 28 days, P.O.); and group 4: Isch+GSE (the common carotid arteries were cut bilaterally and received GSE 100 mg/kg, 28 days, P.O.).

***GSE preparation***

Grape fruits (*Vitis vinifera *L) as large clusters with red barriers were purchased from grape gardens, Qazvin-Iran. Seeds were removed from the grapes, air dried in shade for one week and milled to obtain fine powder (electric mill, Panasonic Co. Japan). The seeds powder was macerated in 75% ethanol for 72 hr at room temperature. The ethanolic extract was evaporated (Rotary Evaporator, Heidolph Co. Germany) to eliminate ethanol and obtain GSE as a lyophilized powder (yield 25-30%) (19-20). 

***Surgical procedure***

Cechetti’s method (2010) with little modification was employed. In summary, animals were anesthetized with a mixture of ketamine/xylazine (50/5mg/kg, intraperitoneal). In groups 1 and 4 (Isch+Veh and Isch+GSE), both common carotid arteries were concomitantly occluded by upper and lower ligatures (2-0 silk suture) and subsequently cut bilaterally. In groups 2 and 3 (Sham+GSE and Sham+Veh), the same operation was carried out while carotid arteries remained intact (9). Behavioral assessment, such as sensorimotor (spontaneous activity and symmetry in the movement of four limbs) and gait performance tests were made to prove the ischemic brain damage in all groups of animals one day before (baseline) and 5 days after the operation by an examiner who was blind to the type of surgical procedure. CHI rats with the minimum score of 3 in each test were selected in this study.

***Sensorimotor evaluations***

Sensorimotor evaluations consisted of two tests that have been developed and described by Garcia (21) with some modifications: 

*Spontaneous activity*

Each animal is observed for 5 min in its normal cage. Scores indicate the following: (1) rat moves around, explores the environment and approaches at least three walls of the cage; (2) rat moves around in the cage, without approaching all sides and hesitates to move, although it eventually reaches at least one upper rim of the cage (height=10 cm); (3) rat dose not rise up at all and barely moves in the cage; (4) rat has no movements. 

*Symmetry in the movement of four limbs*

The rat is held in the air by the tail to observe symmetry in the movement of the four limbs. Scores indicate the following: (1) all four limbs extend symmetrically; (2) limbs on one side extend less or more slowly than the other side; or slow extension of the four limbs; (3) limbs on one or both sides show minimal movements; (4) forelimbs on one or both sides do not move at all.

At the end of each evaluation, the sums of the two test scores were assigned to each rat. CHI rats with the minimum score of 3 in each test were selected in this study.

***Gait performance evaluation***

This evaluation was carried out using the elevated platform test (22). Each rat was positioned in the beginning of a 5-cm wide, 60-cm long wood bridge suspended between two platforms. Animals were tested for their ability to remain on the bridge during a single 3 min trial. 

**Table 1:** Sensorimotor scores in different groups receiving either Grape Seed Extract (100 mg/kg, orally) or its vehicle for 28 days

Groups	Spontaneous activity	Symmetry in the movement of four limbs	Length of the bridge covered	No. of falling
Sham+Veh	3.1±0.1	3.5±0.05	3±0.01	0.3±0.02
Sham+GSE	3.1±0.1	3.5±0.05	3±0.01	0.3±0.02
Isch+Veh	1±0.1^**^	1±0.1^**^	0.5±0.01^**^	4.75±0.3^**^
Isch+GSE	2.08±0.1^##^	1.5±0.05	2±0.01^#^	2.08±0.1^##^

Data are presented as mean±SEM, * significance to sham+Veh, # significance to Isch+Veh

Sham+Veh: sham operated received normal saline, Sham+GSE: sham operated treated with 100 mg/kg grape seed extract, Isch+Veh: Ischemic rats received normal saline, Isch+GSE: Ischemic rats treated with 100 mg/kg grape seed extract

The number of rats falling from the bridge and the length of the bridge covered by each animal, either falling or not, was recorded.

***Passive avoidance task***

This procedure was similar to that previously described (23). Briefly, on the first day of experiment, rats were acclimated to the acquisition chamber. On the second day, rats were gently placed on the wooden platform and latency to step-down (SDL) was recorded as learning phase. When all four paws touched the grid, a low-level electric shock (0.3 mA, 3 sec) was delivered. On the days 1, 3, 7 and 14 after shock delivery, the rats’ step-down latencies were measured (maximum 300 sec) while no shock was applied. 

***LTP recording***

Under ketamine/xylazine anesthesia (90/10 mg/kg, IP), a pair of recording microelectrodes (tungsten wire, CFW, USA) was implanted into the left dentate gyrus (DG) at AP:-3.8 [from bregma], ML: -2.2, DV: 3.5 mm from dura. A pair of stimulating microelectrodes (stainless steel wire, CFW, USA) was implanted into ipsilateral perforant path (PP) at AP: -7.5 [from bregma], ML: -4, DV: 3.9 mm from the dura (24). Single monopolar pulses (duration 50 µs) were delivered at 30 sec intervals. The baseline intensity was selected to result in a field excitatory postsynaptic potential (fEPSP) with 40% of its maximum amplitude by input/output (I/O) curve with different intensities. The signal was amplified (_× _1000), filtered (0.1 Hz-3 kHz), digitized at 2 kHz and stored on the computer. High-frequency stimulation (HFS) to induce LTP consisted of six trains of 6 pulses (50 µs) at 400 Hz, 100 ms between each train, that repeated six times at a 20 sec interval (25). *In vivo* LTP was recorded during 1, 3, 24, and 48 hr after HFS, respectively. Amplitude, slope, and under curve area of population spike (PS) were measured. The recorded PS was analyzed as percentage increase of baseline field excitatory postsynaptic potential (fEPSP). 

***Statistical analysis***

Data were expressed as mean±SEM Step-down latencies at 1^st^, 3^rd^, 7^th^, and 14^th^ days of retention trials were analyzed by one-way ANOVA and properties of recorded PS were analyzed by repeated measured two-way ANOVA, followed by HSD as well as LSD *post hoc *tests. The statistical significance was considered as *P*<0.05. 

**Figure 1:**
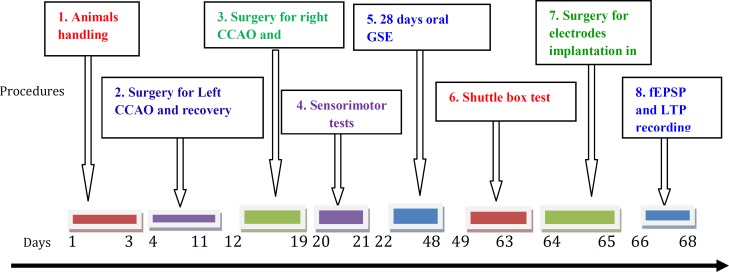
An arrow chart to show different stages of experimental procedures from day 1 to day 68 in each experimental group

## Results

***Sensorimotor activities***

As shown in table 1, the spontaneous activity and symmetry in the movement of four limbs, length of the bridge covered, and number of falls was evaluated during 3 min in all groups. Mean±SEM of spontaneous activity and symmetry in the movement (3.1±0.1 and 3.5±0.05, respectively) in Isch+Veh group were significantly decreased (*P*<0.01) in comparison with Sham+Veh group scores in same examinations (1±0.1 and 1±0.1). The length of the bridge covered performances in Isch+GSE group (2±0.01) was significantly increased (*P*<0.01) compared with Isch+Veh (0.5±0.01). Number of falls in Isch+GSE (2.08±0.1) was significantly increased (*P*<0.01) when compared with Isch+Veh (4.75±0.3). 

***Passive avoidance memory***

**Figure 2:**
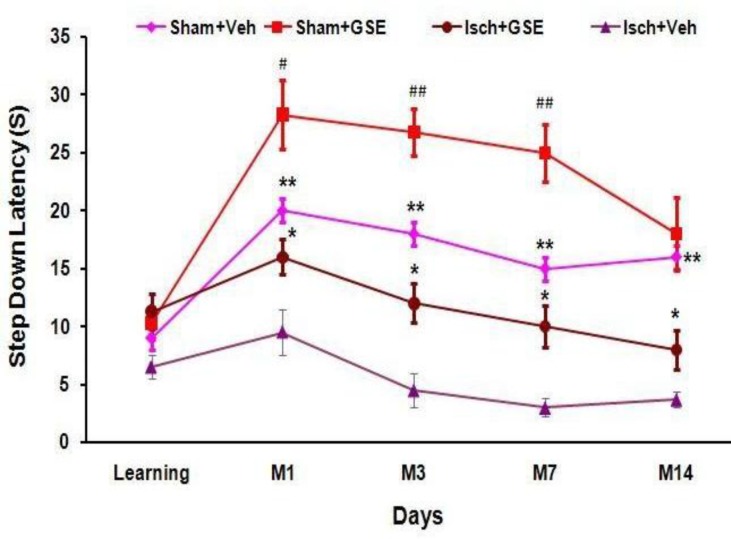
Mean±SEM of step-down latency (SDL) in Sham+Veh, Sham+GSE, Isch+GSE, Isch+Veh groups during learning and memory on different days (**P <0.01, Isch+Veh vs. Sham+Veh; *P<0.05, Isch+GSE vs. Isch+Veh; #*P*<0.05 and ##* P* <0.01, Sham+GSE vs. Sham+Veh, one way ANOVA followed by LSD *post hoc* test, n=8). Sham+Veh: sham operated received normal saline, Sham+GSE: sham operated treated with 100 mg/kg grape seed extract, Isch+Veh: Ischemic rats received normal saline, Isch+GSE: Ischemic rats treated with 100 mg/kg grape seed extract

Step-down latency (SDL) was significantly decreased during memory trials on the 1^st^, 3^rd^, 7^th^, and 14^th^ day after shock delivery to foot paw in Isch+Veh group (rats with permanent 2CCAO received normal saline) when compared with Sham+Veh (*P*<0.01). SDL was significantly increased in Isch+GSE group in comparison with Isch+Veh (*P*<0.05). Moreover, it increased significantly in Sham+GSE group when compared to Sham+Veh (*P*<0.05, *P*<0.01). There were no significant differences between all groups during learning phase (Figure 2).

***Electrophysiology***

**Figure 3:**
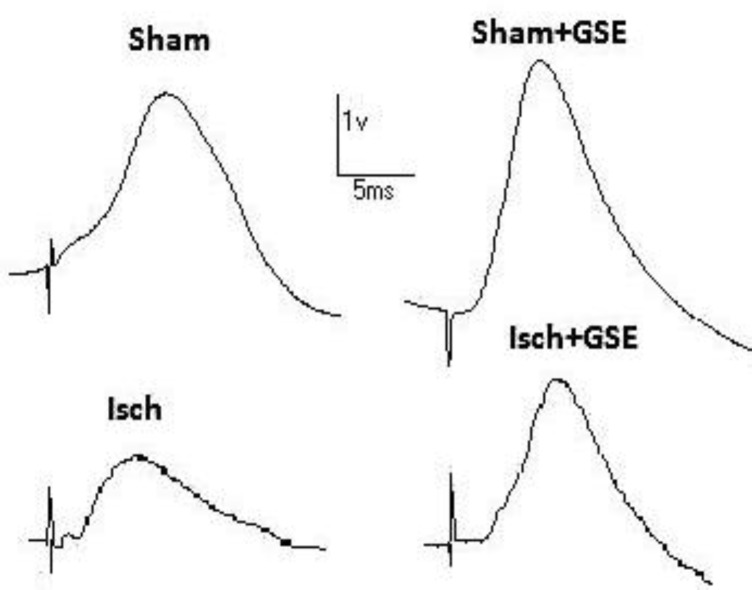
fEPSP traces recorded after LTP induction in different groups Sham: sham operated, Sham+GSE: sham operated treated with 100 mg/kg grape seed extract, Isch: Ischemic rats, Isch+GSE: Ischemic rats treated with 100 mg/kg grape seed extract

**Figure 4:**
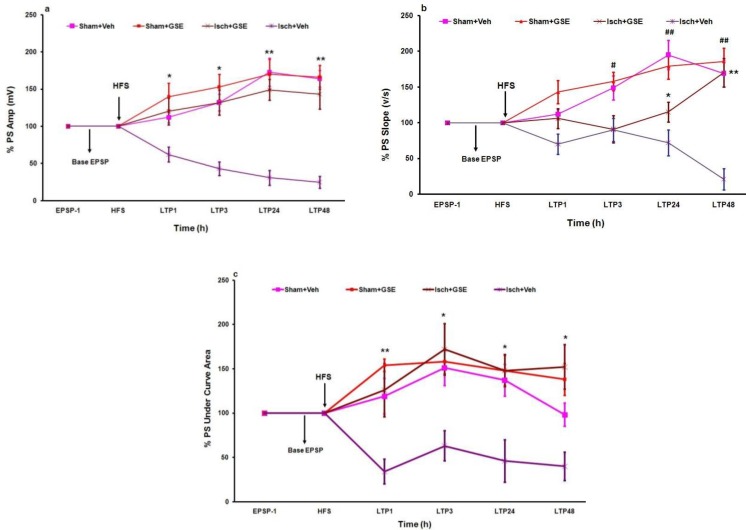
Mean±SEM of percentages of amplitude (a), slope (b) and area under curve (c) of population spikes (PS) in different groups during basal fEPSP and LTP recorded from hippocampal dentate gyrus (DG) at 1^st^, 3^rd^, 24^th^, and 48^th^ hrs after high frequency stimulation (HFS) to brain perforant path (PP). (Repeated measures two-way ANOVA, followed by LSD *post hoc *test, n=8, * P <0.05 and ** P <0.01, Isch+GSE and other groups vs. Isch+Veh, * P<0.05 and **P<0.01, Isch+GSE vs. Isch+Veh; #* P* <0.05 and ##* P* <0.01, Sham+Veh vs. Isch+Veh). Sham+Veh: sham operated received normal saline, Sham+GSE: sham operated treated with 100 mg/kg grape seed extract, Isch+Veh: Ischemic rats received normal saline, Isch+GSE: Ischemic rats treated with 100 mg/kg grape seed extract. EPSP-1: EPSP recorded one hour before high frequency stimulation (HFS), LTP1, LTP3, LTP24 and LTP48: LTP recorded during different times after HFS

Examples of LTP recorded from DG are shown in Figure 3. Animals were subjected to HFS and displayed LTP data. Oral administration of GSE for 28 consecutive days in rats with permanent 2CCAO resulted in a significant increase in the PS amplitude and area under curve (AUC) (Figure 4a & c), whereas significant increase appeared in slope 24 and 48 hours after HFS (*P<0.05, **P<0.01, Figure 4b). fEPSP and PS in Isch+Veh group were significantly lower than all other experimental groups. There were no significant differences in amplitude and area under curve (AUC) between Sham+Veh, Sham+GSE and Isch+GSE groups (Figure 3a & c). 

## Discussion

Our findings showed that chronic oral administration of GSE ameliorates the passive avoidance memory deficit induced by 2CCAO, manifested in longer step-down latency. Moreover, GSE increased percentage of amplitude, slope, and AUC of LTP recorded from hippocampal DG after HFS. Furthermore, 2CCAO weakens the PS recorded from hippocampus DG subdivision that is consistent with a number of previous findings by other researchers (10, 16). 

Cerebral ischemic stroke is a neurological disease where the neuronal cell death happens due to series of pathophysiological events such as energy failure, excitotoxicity, oxidative stress, inflammation, and apoptosis. Therefore, it is called ‘ischemic cascade’ (26). Ischemia causes acute necrotic death in the "core" of the ischemic area leading to resting membrane potential disruption and neuronal swelling (27-28). Thus, subjects suffering from CHI are incapable of learning and storing new experiences since many neurons in critical areas of the brain are dead or utterly damaged. 

 Hippocampus, as a critical area of the brain involved in cognitive function, is very sensitive to ischemia (10). In addition, it has been reported that some parts of the tissue are characterized by a low capillary density compared with the neighboring subdivisions (29). Pyramidal neurons in the CA1 region of the hippocampus are particularly vulnerable and become dead after global ischemia, and therefore hippocampal CA1 injury is observable a few days after untreated forebrain ischemia in rat (3, 5-7, 30), gerbil and human (7, 9). Accordingly, cerebral ischemia could weaken and disrupt the synaptic transition and hippocampal PS in animals as well as humans (31). 

Oxidative stress leads to generation of reactive oxygen species (ROS) within the brain tissue during HI and plays an important role in the development of cerebral damage (32). These oxygen species are extremely reactive and attack lipids, proteins, and nucleic acids, which eventually results in tissue injury and cell death (33). 

It has been established that the major mechanism involved in neurodegeneration following stroke and brain trauma is an increase in NMDA glutamate receptor density and excessive Ca^2+^ influx in the hippocampus (16). Moreover, it has been reported that glutamate (Glu) release in the brain tissue was increased following cerebral ischemia (34). This ischemia-induced release of glutamate is likely to occur in human as well (35), and possibly underlies selective damage to the human hippocampus. Glutamate may cause ischemic neuronal death by acting at excitatory NMDA receptors (36) which plays an important physiological role in long-term potentiation and memory formation (37). Thus, the high concentration of NMDA excitatory receptors on the dendrite trees of hippocampal CA1 pyramidal cells probably explains the long-known selective vulnerability of the CA1 zone of the hippocampus to ischemic brain damage (38). Therefore, any drugs or plant materials with ability to decrease the excitatory neurotransmitter levels or its activity in the brain after CHI could have therapeutic potential to improve the consequences and outcome of ischemic conditions (39).

GSE, as a potent antioxidant with inhibitory effects of free radicals, protects damaged neurons (10, 40). It has been reported that GSE with polyphenols and proanthocyanidins (PA) act directly as antioxidants by scavenging reactive oxygen species (41-42). It reduces 8-iso prostaglandin F2α and proapoptotic protein c-jun in cerebral cortex after hypoxia/ischemia (HI). These identified proteins may mediate the neuroprotective actions of GSE (11), while it is claimed that GSE has no effect on memory and motor activities (43). Some reports have shown that active constituents in grape seed extract such as proantocyanidin inhibits glutamate-induced cell death through inhibition of calcium signals and nitric oxide formation in cultured hippocampal neurons of rat (47). In addition, Charradi *et al* demonstrated that GSE could alleviat high fat diet-induced inhibition of glutamine synthetase activity; hence acting as a glutamine-glutamate GABA cycle modulator in the brain (48). Therefore, the protective effect of GSE against CHI-induced memory deficits and LTP inhibition might be partly due to antioxidant and antagonistic effects of GSE on glutamate activities in the brain. 

## Conclusion

Promising effects of GSE might be attributable partly to its antioxidant capacity and antagonistic effect of GSE on glutamate activities in the brain. Nevertheless, further studies are required to establish the potential use of GSE in CHI-induced cognitive impairment.
